# Effect of health literacy on the quality of life of older patients with long-term conditions: a large cohort study in UK general practice

**DOI:** 10.1007/s11136-017-1775-2

**Published:** 2018-01-10

**Authors:** Maria Panagioti, Suzanne M. Skevington, Mark Hann, Kelly Howells, Amy Blakemore, David Reeves, Peter Bower

**Affiliations:** 1 NIHR School for Primary Care Research, Manchester Academic Health Science Centre, University of Manchester, Oxford Road, Manchester, M13 9PL UK; 2 Division of Psychological Sciences and Mental Health, Manchester Centre for Health Psychology and International Hub for Quality of Life Research, University of Manchester, Oxford Road, Manchester, M13 9PL UK; 3 Division of Nursing, Social Work and Midwifery, School of Health Sciences, Manchester Academic Health Science Centre, University of Manchester, Oxford Road, Manchester, M13 9PL UK

**Keywords:** Health literacy, Quality of life, Long-term conditions, Older adults

## Abstract

**Purpose:**

The levels of health literacy in patients with long-term conditions (LTCs) are critical for better disease management and quality of life (QoL). However, the impact of health literacy on QoL in older adults with LTCs is unclear. This study examined the association between health literacy and domains of QoL in older people with LTCs, investigating key socio-demographic and clinical variables, as confounders.

**Methods:**

A prospective cohort study was conducted on older adults (*n* = 4278; aged 65 years and over) with at least one LTC, registered in general practices in Salford, UK. Participants completed measures of health literacy, QoL, multi-morbidity, depression, social support, and socio-demographic characteristics. Multivariate linear regressions were performed to examine the effects of health literacy on four QoL domains at baseline, and then changes in QoL over 12 months.

**Results:**

At baseline, poor health literacy was associated with lower scores in all four QoL domains (physical, psychological, social relationships and environment), after adjusting for the effects of multi-morbidity, depression, social support and socio-demographic factors. At 12-month follow-up, low health literacy significantly predicted declines in the physical, psychological and environment domains of QoL, but not in social relationships QoL.

**Conclusions:**

This is the largest, most complete assessment of the effects of health literacy on QoL in older adults with LTCs. Low health literacy is an independent indicator of poor QoL older patients with LTCs. Interventions to improve health literacy in older people with LTCs are encouraged by these findings.

## Introduction

The management of long-term conditions (LTCs) is a key challenge facing healthcare systems worldwide as the number of people experiencing one or more LTCs rises [1–3]. Care costs of LTCs are high, and are not only steep for patients but also for their families and healthcare systems [4, 5]. Given this resource burden, a key focus of the management of LTCs is to maintain and improve the quality of life (QoL) of patients [5, 6] which is linked with lower rates of unplanned healthcare utilization and costs [5, 7, 8]. Contemporary QoL assessments gather information about how individuals subjectively rate their own well-being, and more specifically on the physical, psychological, and social dimensions of QoL which can be seriously impaired by LTCs [9, 10]. Self-reported QoL in people with LTCs has received increasing attention; it is a good indicator of patients’ capacity to engage in daily activities, and is associated with reduced healthcare utilization rates and costs [11]. Furthermore, certain patient groups who have socio-economic deprivation, poor education, limited health literacy, symptoms of depression and little social support are more likely to receive sub-optimal quality of care for these conditions, and to report poor QoL [12–14].

Health literacy is defined as ‘*the degree to which individuals have the cognitive and social skills to appropriately access, understand and use health information and services to maintain good health*’ [15]. Health literacy is an indicator of good quality care for LTCs [16] because LTC management requires that patients commit to prolonged therapy [17] and to properly understand the health information to actively participate in their own care [18]. Consistent with this, health literacy has been associated with poor health and critical health outcomes, such as medication adherence and self-management capacity [19–21]. The main advantage of focusing on health literacy is that unlike socio-demographic characteristics which are fixed or difficult to alter, there is some evidence that health literacy can be modified [22]. Enhancing health literacy is important for improving the self-care ability of people with LTCs, and delivering effective, patient-centred, and efficient healthcare.

Over a dozen studies have linked low health literacy with low quality of life (QoL) in people with LTCs [23–35]. Although these studies offer useful insights, they have a number of methodological flaws that compromise their comparability, and raise questions about the generalisability of their results. First, they mainly focus on people with one specific LTC, such as cancer, chronic obstructive pulmonary disease or cardiovascular diseases, and fail to take into account that the presence of multiple LTCs (multi-morbidity) is highly prevalent in older populations [23–28, 31–33, 35]. A second, related limitation is that the majority of these studies used condition-specific tools to measure QoL, which often focus on clinical correlates of that condition, and ignoring the general functioning impairment, and/or the impact of other co-existing LTCs [25–28, 31, 33, 35]. Third, none of these studies focus on patients over aged 65, who are most likely to experience LTCs, and may also face barriers to being literate about health due to limited childhood education, from less access to internet/mobile technology, and diminishing social networks. Fourth, previous studies are mainly cross-sectional, often based on small to medium sample sizes, or show incomplete adjustment of confounders in multivariate analyses [23–27, 30–35]. There is some evidence of that health literacy is associated with QoL, after controlling for socio-demographic factors (e.g. education, age, gender and living/economic conditions, but this evidence is inconsistent) but the influence of other common clinical and emotional factors which determine QoL (e.g. number of LTCs, depression, and perceived support) has not been fully evaluated in this context [2, 7, 36].

The main aim of this study was to examine the effects of health literacy on different domains of QoL, in a large sample of older adults with LTCs, and to do this with cross-sectional and prospective data. We hypothesized that patients with low health literacy levels would report lower QoL at baseline. Furthermore, that health literacy at this time would predict changes in QoL, 12 months later. To examine whether the effects of health literacy on QoL are confounded by other factors, we included as covariates in the analyses several pre-specified socio-demographic and health variables, namely age, gender, education, living status, multiple deprivation index, depression, number of LTCs, and perceived social support.

## Methods

### Participants and recruitment

This study analyses data collected in a large longitudinal cohort study; the Comprehensive Longitudinal Assessment of Salford Integrated Care (CLASSIC). As described in another recent study published from CLASSIC [37], participants in CLASSIC had to meet two main inclusion criteria: (i) aged 65 or over, and (ii) being registered as having at least one long-term condition, with a general practice in Salford, North West England. Individuals receiving palliative care, and those with dementia, were excluded from this study. CLASSIC assessed the results of the Salford Integrated Care Programme which focused on promoting independence of older people by providing access to (a) a local community assets for independent living; (b) an integrated contact centre for navigation, support and co-ordination; (c) multidisciplinary groups for supporting high-risk patients.

Salford has 294,916 (34,000 aged over 65 years) habitants and 52 general practices. Life expectancy in Salford is lower than the national life expectancy and the levels of long-term illness and socio-economic deprivation are higher than the national average. We invited all 52 general practices in Salford to participate in this study. Thirty-three (65%) practices consented to take part. A list of potentially eligible participants was identified in each participating practice, using the FARSITE software (http://nweh.co.uk/products/farsite). Afterwards, we liaised with each practice to identify any patients who met the study exclusion criteria. No incentives were offered to practices, but support costs were provided as reimbursement to practices for time spent checking the patient list generated by FARSITE [37].

12, 989 patients were eligible for participation. The first questionnaire was posted to all participants between November 2014 and February 2015. Reminder letters were sent to non-responders 3 weeks later. The CLASSIC questionnaire mainly included measures of demographic and clinical characteristics (e.g. types of long-term conditions) and validated measures of well-being (symptoms of depression) and quality of life, social support and the user/patient experience of health services. Completion of the questionnaire was anticipated to last approximately 15–20 min. Participants were reimbursed with a £10 voucher after the completed baseline questionnaire was received. Follow-up questionnaires were sent 6 and 12 months later but in this study we report the 12-month data.

### Dependent variables

Quality of life (QoL): A World Health Organisation (WHO) international collaboration developed and standardized a cross-cultural measure in 15 cultures simultaneously, the World Health Organization Quality of Life Assessment (WHOQOL-BREF) short-form instrument [38]. This measure was designed for use by adult populations with chronic diseases and conditions, and well people, and has been validated in UK [7]. This 26-item measure [10] includes two general items on overall QoL and health, and 24 items representing specific QoL facets, and scored in one of the four QoL domains: physical, psychological, social relationships and environmental QoL. The Physical Health domain includes questions in relation to sleep, energy, mobility, the extent to which pain prevents performance of necessary tasks, the need for medical treatment to function in daily life and capacity for work. The Psychological domain includes questions in relation to concentration capacity, self-esteem, body image and mood. The Social Relationships domain includes questions in relation to satisfaction with personal relationships, social support and sex. The Environment domain includes questions in relation to safety and security, physical environment satisfaction and finance [39]. Facet items are rated on a scale from 1 to 5. Raw domain scores range from 4 to 20, and are transformed onto a scale from 0 to 100. Quality of life is assessed over the past 2 weeks.

### Independent variables

#### Socio-demographic characteristics

Age, gender, employment and degree qualifications were assessed using questions derived by the General Practice Patient Survey [40]. We also collected information on living status (alone or with a partner), and ethnicity which was coded according to the 17 categories from the 2011 Census.

#### Health literacy

Health literacy was assessed using the Single Item Literacy Screener (SILS) (rated from 1 = never to 5 = always) [41]: ‘*How often do you need to have someone help you when you read instructions, pamphlets, or other written material from your doctor or pharmacy*?’ This measure has been previously used by people with LTCs, and in addition to feasibility and acceptability demonstrates good reliability and validity [41, 42]. SILS is moderately correlated with other short and more comprehensive measures of health literacy (correlation coefficients r ranging from 0.50 to 0.60; *p* < 0.001 for all correlation coefficients) [50].

#### Long-term conditions

A validated questionnaire assessed the self-reported number and burden of long-term conditions [43]. This questionnaire contains 21 common long-term conditions, and allows patients to report additional conditions not listed. Participants rate each condition on a five-point scale, which assesses level of interference with the daily activities. The total burden score is the sum of conditions weighted by the level of interference assigned to each [43].

#### Depression

The presence of depression was assessed using the Mental Health Inventory (MHI-5); a 5-item scale incorporating questions on general mental health in the past month, including depression, anxiety, behavioural-emotional control and general positive affect [44]. This well-validated measure can identify depressive symptoms, with higher scores indicating better mental health [45, 46].

#### Social support

Social support was assessed using the ENRICHD Social Support instrument (ESSI), a 7-item scale measuring tangible help and emotional support from others, including partners [47]. A total score is the sum of all items. Higher scores indicate better social support.

### Data analysis

Descriptive statistics of all variables in the analyses were calculated. All analyses focused on cases with valid scores for the QoL domains after the manual scoring rules were applied. Imputations were performed for independent variables with missing values. Regression imputations (linear, binary logistic, ordinal logistic or multinomial as appropriate) were generated for each independent variable with missing values, using the other independent variables and the baseline scores of the dependent variables, as predictors. Following the imputation process, *n* = 568 cases were added in the analyses for all domains. The analyses were performed with, and without imputed cases, to examine the validity of the imputations. The results were similar and therefore we present only the analyses following imputations.

The variance inflation factors (VIFs) values were inspected to assess level of multicollinearity between explanatory variables. VIFs amongst the explanatory variables entered into all multivariate analyses were below 10, indicating acceptable multicollinearity. Correlations and paired t tests were performed to examine associations between QoL domains at baseline, and follow-up. Normality was examined using Shapiro–Wilk tests. These tests were statistically non-significant for all QoL domains at baseline, and follow-up suggesting that data were normally distributed.

Two multivariate multiple regression analyses were conducted [48]. The first analysis explored relationships between explanatory variables and QoL domain scores at baseline. The second analysis repeated this, but used as the dependent variables, the calculated difference in each QoL domain score from baseline to 12-month follow-up (change scores). Our prospective analysis focused on change scores because this approach is less biased than the analysis of outcome variables when using observational data [49]. Raw and standardized regression coefficients and R^2^ (raw and adjusted) values are reported. All analyses were undertaken using Stata (version 14).

## Results

### Descriptive characteristics

**Fig. 1:**
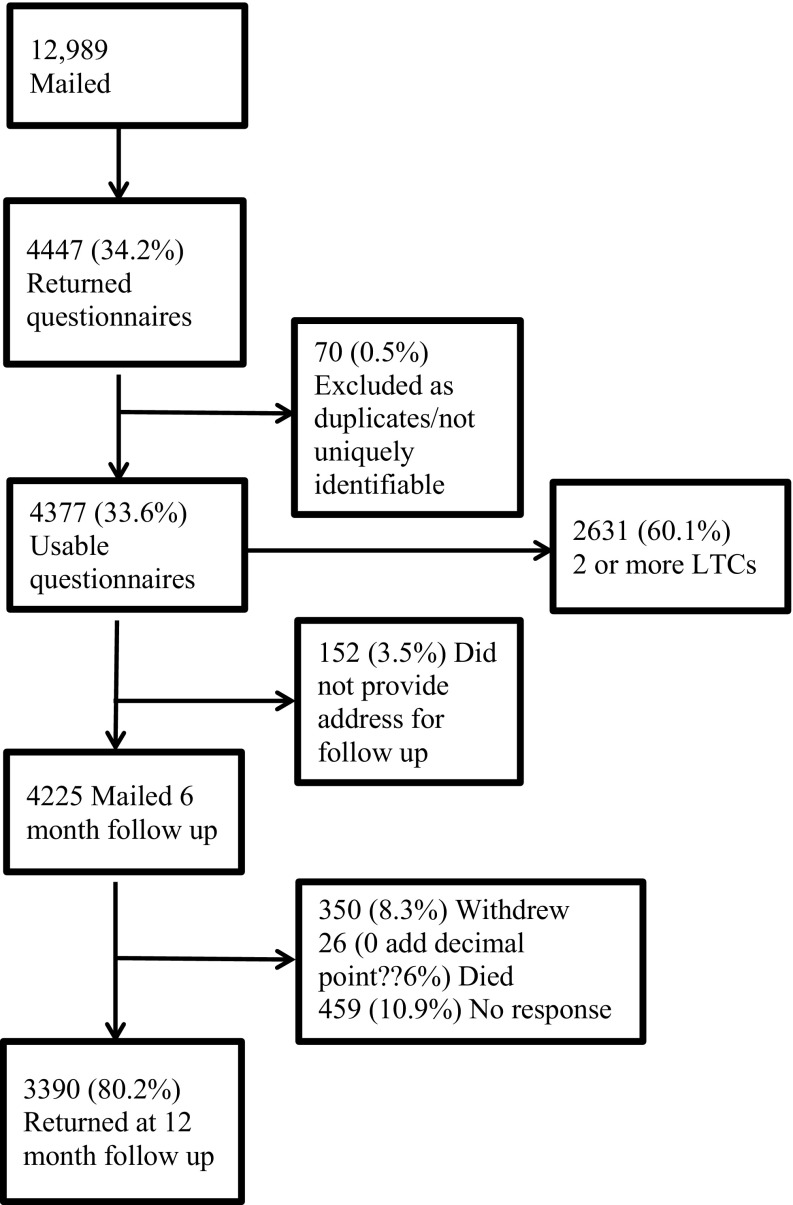
Flow of participant selection

As shown in the flow diagram (Fig. 1) 33% of the eligible participants returned the questionnaire at baseline (*n* = 4377 out of 12,989). At 12-month follow-up, 77% returned the questionnaires of those mailed (*n* = 4225 out of 3242).

The participants in this study were mainly white British retired women (see Table 1). The total number of cases following imputations was 3760 (89% of those returned the questionnaires) at baseline, and 2706 (83% returned questionnaires) at 12-month follow-up. Approximately 1 in 5 patients reported health literacy problems at least occasionally (i.e. sometimes to always). Multi-morbidity was common in the sample; about 60% reported having 2 or more LTCs.

**Table 1:** Participant characteristics by quality of life subscales from the WHOQOL-BREF

	Physical QoL	Psychological	Environment	Social
Mean age (SD)	74.49 (6.75)	74.50 (6.75)	74.53 (6.77)	74.46 (6.75)
Mean depression (SD)	67.25 (22.39)	67.30 (22.40)	67.31 (22.39)	67.46 (22.36)
Mean LTCs (SD)	2.21 (1.73)	2.21 (1.73)	2.21 (1.73)	2.20 (1.73)
Mean social support (SD)	27.62 (6.99)	27.60 (7.00)	27.58 (7.01)	27.66 (6.97)
Gender				
Male	1855 (47.41)	1866 (47.41)	1866 (47.4)	1861 (48.05)
Female	2058 (52.59)	2070 (52.59)	2071 (52.6)	2012 (51.95)
Living status				
Cohabitating	2532 (64.71)	2539 (64.51)	2544 (64.62)	2533 (65.4)
Living alone	1381 (35.29)	1397 (35.49)	1393 (35.38)	1340 (34.6)
Ethnicity				
White	3837 (98.06)	3859 (98.04)	3862 (98.09)	3799 (98.09)
Non White	55 (1.41)	56 (1.42)	54 (1.37)	55 (1.42)
Degree qualifications				
Yes	2007 (51.29)	2011 (51.09)	2012 (51.1)	2006 (51.79)
No	1906 (48.71)	1925 (48.91)	1925 (48.9)	1867 (48.21)
Employment				
Working	227 (5.82)	194 (4.93)	194 (4.93)	196 (5.06)
Retired	3639 (93.03)	3712 (94.11)	3712 (94.2)	3642 (99.25)
Health literacy				
Never	2787 (71.22)	2794 (70.99)	2798 (71.07)	2755 (71.13)
Rarely need help	386 (9.86)	395 (10.04)	390 (9.91)	396 (10.22)
Sometimes	403 (10.31)	406 (10.32)	405 (10.29)	403 (10.41)
Often/always need help	337 (8.61)	341 (8.66)	344 (8.74)	319 (8.24)

Figures are numbers (percentage) of patients unless otherwise specified

Table 2 presents correlations between baseline and follow-up mean scores on the QoL domains. Across the domains, these were 60.0 (SD = 22.3) for physical QoL, 69.6 (SD = 17.7) for psychological, 72.5 (SD = 16.4) for social relationships and 68.4 (SD = 20.2) for environmental QoL. The overall correlation between baseline and follow-up domains scores ranged from 0.71 to 0.82 (*p* < 0.001). There was a significant difference in the scores for every domain from baseline to 12-month follow-up: physical QoL *t* (3759) = 8.4, *p* < 0.001; psychological *t* (3759) = 12.8, *p* < 0.001; social *t* (3759) = 6.6, *p* < 0.001; environmental *t* (3759) = 8.2, *p* < 0.001.

**Table 2:** Summary statistics for outcome measures

Quality of life domains	Baseline (*N*)	Baseline mean (SD)	Follow-up (*N*)	Mean (SD)	Correlation: baseline and follow-up
Physical	4131	60.03 (22.31)	3156	60.18 (20.81)	0.82***
Psychological	4159	69.59 (17.73)	3190	68.35 (16.82)	0.77***
Environment	4096	68.41 (20.16)	3117	67.29 (19.57)	0.71***
Social relationships	4173	72.50 (16.39)	3167	72.07 (15.68)	0.75***

***p < 0.001

### Predictors of quality of life domains at baseline

The multiple regression results of baseline data are presented in Table 3.

**Table 3:** Results from multivariate regression analyses of quality of life domains at baseline

	Physical QoL			Psychological QoL			Environment QoL			Social QoL		
	*b* (95% CI)	Beta	*p*	*b* (95% CI)	beta	*p*	*b* (95% CI)	beta	*p*	*b* (95% CI)	beta	*p*
Age	− 0.23 (− 0.31 to − 0.14)	− 0.07	0.000	0.00 (− 0.06 to 0.06)	0.00	0.967	− 0.09 (− 0.16 to − 0.03)	− 0.04	0.004	0.20 (0.11–0.28)	0.07	0.000
Gender												
Male	Ref			Ref			Ref			Ref		
Female	0.09 (− 0.96 to 1.14)	0.00	0.866	− 0.39 (− 1.15 to 0.38)	− 0.01	0.321	0.96 (0.16–1.76)	0.03	0.019	7.26 (6.22–8.29)	0.18	0.000
Qualifications												
Yes	Ref			Ref			Ref			Ref		
No	− 1.45 (− 2.54 to − 0.37)	− 0.03	0.009	− 0.44 (− 1.23 to 0.35)	− 0.01	0.271	− 2.08 (− 2.91 to − 1.25)	− 0.06	0.000	0.20 (− 0.87 to 1.28)	0.01	0.710
Living status												
Living alone	Ref			Ref			Ref			Ref		
Cohabitating	1.98 (0.65–3.31)	0.04	0.003	3.05 (2.09–4.02)	0.08	0.000	3.74 (2.73–4.75)	0.11	0.000	5.15 (3.82–6.47)	0.12	0.000
Employment												
Retired	Ref			Ref			Ref			Ref		
Employed	3.87 (1.73–6.00)	0.04	0.000	2.73 (1.17–4.30)	0.04	0.001	0.29 (− 1.35 to 1.94)	0.00	0.725	1.40 (− 0.71 to 3.52)	0.02	0.193
Health literacy												
Never	Ref			Ref			Ref			Ref		
Rarely	− 5.34 (− 7.10 to − 3.59)	− 0.07	0.000	− 2.82 (− 4.09 to − 1.55)	− 0.05	0.000	− 4.90 (− 6.24 to − 3.56)	− 0.09	0.000	− 1.88 (− 3.60 to − 0.17)	− 0.03	0.032
Sometimes	− 7.75 (− 9.53 to − 5.96)	− 0.11	0.000	− 6.39 (− 7.68 to − 5.09)	− 0.11	0.000	− 7.03 (− 8.39 to − 5.67)	− 0.13	0.000	− 1.61 (− 3.37 to 0.15)	− 0.02	0.073
Often/always	− 13.6 (− 15.64 to 11.60)	− 0.17	0.000	− 10.5 (− 11.93 to − 8.99)	− 0.17	0.000	− 9.8 (− 11.32 to − 8.24)	− 0.17	0.000	− 4.80 (− 6.85 to − 2.76)	− 0.07	0.000
LTCs	− 4.50 (− 4.81 to − 4.18)	− 0.35	0.000	− 1.05 (− 1.28 to − 0.82)	− 0.10	0.000	− 1.27 (− 1.51 to − 1.02)	− 0.13	0.000	− 1.18 (− 1.50 to − 0.87)	− 0.10	0.000
Depression	0.34 (0.31–0.36)	0.34	0.000	0.44 (0.42–0.46)	0.55	0.000	0.24 (0.22–0.26)	0.33	0.000	0.25 (0.23–0.28)	0.28	0.000
Social support	0.17 (0.08–0.26)	0.05	0.000	0.56 (0.50–0.63)	0.22	0.000	0.73 (0.66–0.80)	0.31	0.000	1.30 (1.21–1.39)	0.45	0.000
IMD	− 0.08 (− 0.11 to − 0.05)	− 0.07	0.000	− 0.01 (− 0.03 to 0.01)	− 0.01	0.513	− 0.10 (− 0.12 to − 0.07)	− 0.11	0.000	− 0.04 (− 0.07 to − 0.01)	− 0.03	0.012

*Ref* reference category in the analysis

#### Physical QoL

Older age, poor health literacy, higher multiple deprivation scores, being retired, having no degree qualification, more severe depressive symptoms and a greater number of LTCs were associated with significantly lower physical QoL scores at baseline. Living with a partner and better social support had a positive effect on physical QoL. The regression model as a whole, explained a total 48.3% of the variance in physical QoL. The strongest predictors of physical QoL at baseline were number of LTCs and health literacy.

#### Psychological QoL

Being retired, poor health literacy, more depressive symptoms and a greater number of LTCs were associated with significantly lower QoL scores in the psychological QoL at baseline. Living with a partner had a positive effect on psychological QoL. Overall the regression model explained 56.4% of the variance in the psychological QoL domain. The strongest predictors of psychological QoL at baseline were health literacy and living with a partner.

#### Environment QoL

Older age, having no degree qualification, poor health literacy, severe depressive symptoms and a greater number of LTCs were associated with significantly lower environmental QoL scores, at baseline. Living with a partner and having better social support had a positive effect on environmental QoL. Overall the regression model explained 42.9% of the variance in the environment domain. The strongest predictors of environment QoL at baseline were living with a partner, health literacy and number of LTCs.

#### Social relationships QoL

More severe depressive symptoms, poor health literacy and a greater number of LTCs, were associated with significantly lower scores on the social relationships domain at baseline. Being older and female, living with a partner and having better social support were associated with higher social QoL. The regression model as a whole, explained 38.3% of the variance in the social relationships domain. The strongest predictors of social QoL at baseline were being female, living with a partner, health literacy and social support.

### Predictors of change in quality of life domains over 12 months

The results of the multiple regression analyses of changes to QoL between baseline and follow-up are presented in Table 4.

**Table 4:** Results from multivariate regression analyses of changes in quality of life domains at 12-month follow-up

	Physical QoL			Psychological QoL			Environment QoL			Social QoL		
	*b* (95% CI)	Beta	*p*	*b* (95% CI)	Beta	*p*	*b* (95% CI)	Beta	*p*	*b* (95% CI)	Beta	*p*
Age	0.01 (− 0.07 to 0.09)	0.00	0.831	0.01 (− 0.06 to 0.08)	0.01	0.701	− 0.02 (− 0.09 to 0.05)	− 0.01	0.547	0.04 (− 0.05 to 0.14)	0.02	0.362
Gender												
Male	Ref			Ref			Ref			Ref		
Female	0.79 (− 0.15 to 1.74)	0.03	0.100	1.23 (0.38–2.09)	0.05	0.005	0.96 (0.12–1.79)	0.04	0.025	− 0.40 (− 1.57 to 0.76)	− 0.01	0.497
Degree qualifications												
Yes	Ref			Ref			Ref			Ref		
No	− 0.59 (− 1.57 to 0.39)	− 0.02	0.235	− 1.02 (− 1.90 to − 0.14)	− 0.04	0.023	− 0.33 (− 1.19 to 0.54)	− 0.01	0.458	− 0.53 (− 1.74 to 0.68)	− 0.02	0.392
Living status												
Living alone	Ref			Ref			Ref			Ref		
Cohabitating	− 0.15 (− 1.36 to 1.07)	− 0.01	0.810	− 0.49 (− 1.59 to 0.61)	− 0.02	0.383	− 0.58 (− 1.65 to 0.49)	− 0.02	0.290	− 0.68 (− 2.19 to 0.83)	− 0.02	0.379
Employment												
Retired	Ref			Ref			Ref			Ref		
Employed	0.48 (− 1.42 to 2.38)	0.01	0.621	0.69 (− 1.04 to 2.43)	0.01	0.434	0.67 (− 1.02 to 2.35)	0.01	0.438	0.72 (− 1.63 to 3.08)	0.01	0.547
Health literacy												
Never	Ref			Ref			Ref			Ref		
Rarely need help	0.49 (− 1.12 to 2.11)	0.01	0.550	0.26 (− 1.18 to 1.71)	0.01	0.720	− 0.20 (− 1.63 to 1.22)	− 0.01	0.781	0.81 (− 1.16 to 2.77)	0.02	0.422
Sometimes	1.52 (− 0.16 to 3.19)	0.03	0.076	1.84 (0.34–3.34)	0.05	0.016	2.49 (1.02–3.96)	0.07	0.001	0.72 (− 1.34 to 2.78)	0.01	0.492
Often/always	3.74 (1.57–5.92)	0.07	0.001	2.60 (0.65–4.54)	0.05	0.009	3.40 (1.51–5.29)	0.07	0.000	1.69 (− 1.05 to 4.43)	0.02	0.226
Long-term conditions	0.62 (0.32–0.93)	0.08	0.000	− 0.15 (− 0.43 to 0.12)	− 0.02	0.275	− 0.01 (− 0.28 to 0.26)	0.00	0.951	− 0.02 (− 0.39 to 0.36)	0.00	0.937
Depression	− 0.03 (− 0.05 to − 0.01)	− 0.05	0.012	− 0.06 (− 0.09 to − 0.04)	− 0.12	0.000	− 0.02 (− 0.04 to 0.00)	− 0.03	0.098	− 0.01 (− 0.04 to 0.02)	− 0.02	0.444
Social support	0.07 (− 0.01 to 0.16)	0.04	0.082	− 0.02 (− 0.09 to 0.06)	− 0.01	0.635	− 0.11 (− 0.18 to − 0.03)	− 0.07	0.005	− 0.19 (− 0.29 to − 0.09)	− 0.09	0.000
IMD	0.00 (− 0.03 to 0.03)	0.00	0.950	− 0.01 (− 0.03 to 0.02)	− 0.01	0.664	0.01 (− 0.01 to 0.04)	0.02	0.251	0.00 (− 0.03 to 0.04)	0.01	0.799

*Ref* reference category in the analysis

#### Physical health QoL

Changes to QoL in the physical domain were predicted by the severity of depressive symptoms, the number of LTCs and levels of health literacy. Physical QoL declined over the year for those who reported lower levels of health literacy, greater number of LTCs and more severe symptoms of depression previously, at baseline.

#### Psychological QoL

Changes in the psychological domain were predicted by gender, educational level, health literacy and depression symptoms. Psychological QoL declined for those who were male, had no degree qualifications, poor health literacy and increased severity of depressive symptoms, at baseline.

#### Environment QoL

Changes in environmental QoL were predicted by gender, the level of health literacy and social support at baseline. Being male with low health literacy and low social support at baseline, a greater decline is predicted in environmental QoL.

#### Social relationships QoL

Only social support accounted for changes in the social relationships domain over the 12 months of the study. Social relationships QoL declined for those who reported lower levels of social support at baseline.

## Discussion

### Summary of main findings

In this study, we examined the effects of health literacy on QoL domains, while controlling for selected socio-demographic and health-related factors which were previously found to be associated with QoL in this older population. At baseline, poor health literacy was associated with lower QoL, across all four domains (physical, psychological, social relationships and environment), after adjusting for the effects of multi-morbidity, depressive symptoms, social support and socio-demographic factors. Baseline multi-morbidity and severity of depressive symptoms were significantly negatively associated with every QoL domain, whereas social support was positively associated with each QoL domain.

At follow-up, baseline health literacy predicted declines in physical, psychological and environmental QoL, but not social QoL. Severity of depressive symptoms independently predicted declines in physical and psychological QoL, but only multi-morbidity predicted declines in environmental QoL. Social support was the strongest predictor of improvement in social and environmental domains of QoL. However, health literacy, as well as other predictors in the regression models, explained a significant proportion of the variance at baseline, whereas the predictive abilities of change were very small after 1 year, for all the variables in the model.

### Strengths and weaknesses of the study

Major strengths of this study are the use of a large sample of older adults who have poorer health literacy compared to other age groups, the prospective design which allowed the investigation of predictors of change in QoL and the comprehensive assessment of confounders in the analyses.

There are also limitations. The assessment of health literacy in this study is likely to be incomplete and no information is available about the link between QoL and different types of health literacy in people with LTCs. Although SILS is a widely used measure with acceptable correlations with other measures of health literacy [50], which has been used in people with LTCs in the past [41, 42], it can be best as a ‘screening’ instrument for low health literacy rather a full diagnostic instrument for low health literacy. These findings therefore are not definitive but they provide the basis for undertaking additional studies of health literacy in this population using more comprehensive ‘diagnostic’ measures of health literacy. Second, approximately one-third of all eligible patients (34%) agreed to take part in this study. This response rate is comparable to the response rates of previous studies using similar methods and participants [51] but we have no data to ascertain whether those agreed to participate in this study differ from those who did not provide consent to participate. However, completion of follow-up exceeded 80%, indicating good retention, and suggesting a low risk for response bias. Third, the prevalence of characteristics and conditions in the sample, such as the number of LTCs, are based on self-reports, and these values could differ from information held in medical records. Fourth, written consent was sought from the participants of this study. Although this is a common approach in health research, people with poor health literacy may have encountered problems in providing written consent and therefore they might be under-represented in this study. Future studies on health literacy using oral consent would be useful to confirm these findings. Finally, our findings are about the experience of patients with LTCs living in one highly deprived area of the UK, where the population is predominantly ethnically white. There is evidence elsewhere that some ethnic groups report poorer experiences of health care than whites, and it is plausible that they might also have reported lower health literacy and more negative experiences of QoL [52, 53], had they been included.

### Research and theoretical implications

Health literacy impacts on QoL in people with LTCs. This is shown by the increasing number of empirical studies that have investigated this relationship quantitatively [23–35]. However, a key discrepancy remains in the literature; whether there is a direct causal association between health literacy and QoL, or whether this association is explained by poorer physical or mental health (e.g. number/severity of LTCs depression), limited social support or socio-economic deprivation. This study found that health literacy has an independent effect on all dimensions of QoL in older patients with LTCs. The implications therefore underscore the importance of focusing on developing policy to address health literacy challenges faced by this ageing population. Accordingly, research evidence suggests that patients aged 65 and over have the poorest health literacy skills while at the same time face unique physical and cognitive difficulties which limit their ability to access health information compared to other age groups [54]. Other studies mainly on middle-aged adults found that the link between health literacy and QoL can be attributed to the severity of multi-morbidity, self-care capacity, and deprivation measures [26, 32]. It is likely that health literacy interferes more with QoL as people age, but this hypothesis needs to be tested empirically in future longitudinal studies.

The notion that health literacy impacts on QoL outcomes is theoretically supported by generic conceptual models of health literacy,[55] and models focused on specific LTCs such as diabetes [56, 57]. These models postulate that health literacy affects health outcomes, and that behavioural factors like social support, or support for the management of the disease, as well as patient engagement in self-management, play significant roles in this link, either as full or partial mediators. A fruitful future direction would be to confirm the link between health literacy and QoL in multiple follow-up time points, and to examine potential mediators of this relationship, incorporating the effects of behavioural factors (e.g. support and involvement in self-management), health factors (multi-morbidity, depression), and socio-demographic factors (educational level and socio-economic deprivation measures).

Subjective QoL assessments are increasingly used [58], and often indicate that QoL is multidimensional in nature, typically encompassing physical, psychological and social perceptions. An advantage of the WHOQOL instruments is that they also enable environmental QoL perceptions to be assessed [9, 10]. Understanding which aspects of QoL are affected by low health literacy can facilitate efforts to maintain and improve QoL in older adults with LTCs. This study makes an important contribution in this field by differentiating three QoL domains that are associated with health literacy (taking other salient factors into account), from the social domain which seems unaffected. It is not surprising that physical, psychological, and environmental domains are more affected by poor health literacy, as they are closely linked to ill health and the capacity to access healthcare, compared to social QoL which is more linked with the quality of interpersonal relationships and networks. However, this finding might just reflect measurement issues as the social domain of the WHOQOL-BREF contains only three items, which might not entirely capture the significance of effects of low health literacy on this area of QoL. The novel findings on environmental QoL offer a rare contribution from a QoL domain which is rarely assessed in health. Its facets on perceived opportunities to acquire information and skills, access to health and social care, financial resources, home and physical environments, are highly relevant to the lives of older people living in socio-economically deprived areas.

To date, the majority of interventions for improving health literacy have focused on reducing the cognitive demands of the health information available to patients (e.g. increasing the readability of instructions or being assisted by healthcare staff) [59]. Our finding that health literacy is associated with declines in the QoL of older people with LTCs raises the need for testing ways to improve health literacy, rather than just reducing the cognitive demands of available health information.

### Implications for policy and practice

In terms of policy and clinical practice, these findings emphasize the need to ensure that older adults with LTCs can learn about and ultimately access appropriate health and social services to maintain a good QoL. Healthcare clinicians should be aware of the health literacy problems in older adults, to assess the health literacy skills and try to address these problems where possible [60]. Simple approaches such as communicating in plain terms, simplifying health-related information and using interview techniques (e.g. the ‘Teach back technique’) [61] can increase the responsiveness of patients with low health literacy in consultations and self-management plans [62, 63]. Additionally, improving the skills of health literacy trainers to work with older people with LTCs is of critical importance because working with this population is challenging [64]. Multi-faceted and collaborative interventions have the potential to advance the health literacy and independence of people with LTCs [63, 65] but further evidence on the effectiveness and cost-effectiveness of such strategies is needed.

## Conclusions

We found that poor health literacy is a major independent predictor of lower QoL in older patients with LTCs, which is a core outcome for clinical studies. This finding highlights the need to increase awareness by systematically assessing and intervening to reverse poor health literacy in older patients with LTCs. When designing interventions and care plans to improve QoL outcomes in older patients, it is crucial to consider the health literacy levels in this population.
